# Hysteroscopic resection of type 3 fibroids could improve the pregnancy outcomes in infertile women: a case–control study

**DOI:** 10.1186/s12884-022-04828-3

**Published:** 2022-06-28

**Authors:** Ying Han, Ruqiang Yao, Yinfeng Zhang, Zexin Yang, Haining Luo, XinYan Wang, Aijun Du, Yunshan Zhang, Yingjun Zhu

**Affiliations:** 1 Tianjin Key Laboratory of Human Development and Reproductive Regulation, Tianjin Central Hospital of Gynaecology Obstetrics, No 156 Sanma Road, Nankai District, Tianjin, 300100 China; 2 Tianjin Medical University, Tianjin, 300070 China

**Keywords:** Fibroid, Hysteroscopy, In vitro fertilization, Pregnancy, Live birth

## Abstract

**Background:**

Type 3 fibroids are a special subtype of intramural fibroids that are likely to affect the pregnancy outcomes of assisted reproductive techniques. Hysteroscopic resection is a treatment for type 3 fibroids, but there has few study of its efficacy to date. In this study we evaluated the effect of hysteroscopic resection of type 3 fibroids on the pregnancy outcomes in infertile women.

**Methods:**

This retrospective case–control study was conducted from January 1, 2014 to June 30, 2021. Patients who underwent IVF-ICSI in our unit were divided into a type 3 fibroid group and a hysteroscopic myomectomy group. The inclusion criteria for the type 3 fibroid group and the hysteroscopic myomectomy group were as follows: 1) age ≤ 40 years; 2) fibroid diameter or total fibroid diameter > 2.0 cm. The following exclusion criteria were used: 1) oocyte donor treatment cycles and 2) presence of chromosomal abnormalities; 3) history of other uterine surgery; 4) presence of intracavitary lesions, including submucosal fibroids; 5) single fibroid > 5.0 cm; 6) cervical fibroids; 7) unclear ultrasound description of fibroids; 8) preimplantation genetic testing was performed and 9) congenital or acquired uterine malformations. The control group in our study was selected from patients who were treated with IVF only because of fallopian tube factors. According to the age of the type 3 fibroid group and hysteroscopic myomectomy group, random sampling was carried out in the patients between 25 and 47 years of age to determine a control group. The outcomes measured included the average transfer times to live birth, cumulative clinical pregnancy rate, and cumulative live birth rate.

**Results:**

A total of 302 cycles were enrolled in our study, including 125 cycles with type 3 fibroids, 122 cycles with hysteroscopic myomectomy, and 139 cycles of control patients. The average transfer times to live birth were significantly higher in the type 3 fibroid group than in the other two groups. The frequency of cumulative live births in the type 3 fibroid group was significantly lower than that in the control group. Compared with the control group, the hysteroscopic myomectomy patients had no statistically significant differences in the cumulative clinical pregnancy rate and cumulative live birth rate.

**Conclusions:**

Type 3 fibroids significantly reduced the cumulative live birth rate of IVF patients. Ultrasound-guided hysteroscopic myomectomy can be used as a treatment for type 3 fibroids and could improve the pregnancy outcomes in infertile women.

## Introduction

Uterine fibroids are the most common tumours of the female reproductive tract. In recent years, fibroids have attracted attention for their impact on female fertility and pregnancy outcomes, and the cumulative incidence in women of reproductive age is 20% to 50% [1]. Uterine fibroids may be solely responsible for impaired fertility outcomes in 2%–3% of cases [2]. The location of the fibroids, size, and number influence the woman’s fertility and the outcome of pregnancy in unascertained ways [3]. The mechanisms linking uterine fibroids and infertility are numerous: uterine cavity distortion, impaired endometrial and myometrial blood supply, increased uterine peristalsis during mid luteal phase, molecular changes, impaired endometrial receptivity and a thicker capsule [4–7].

**Fig. 1:**
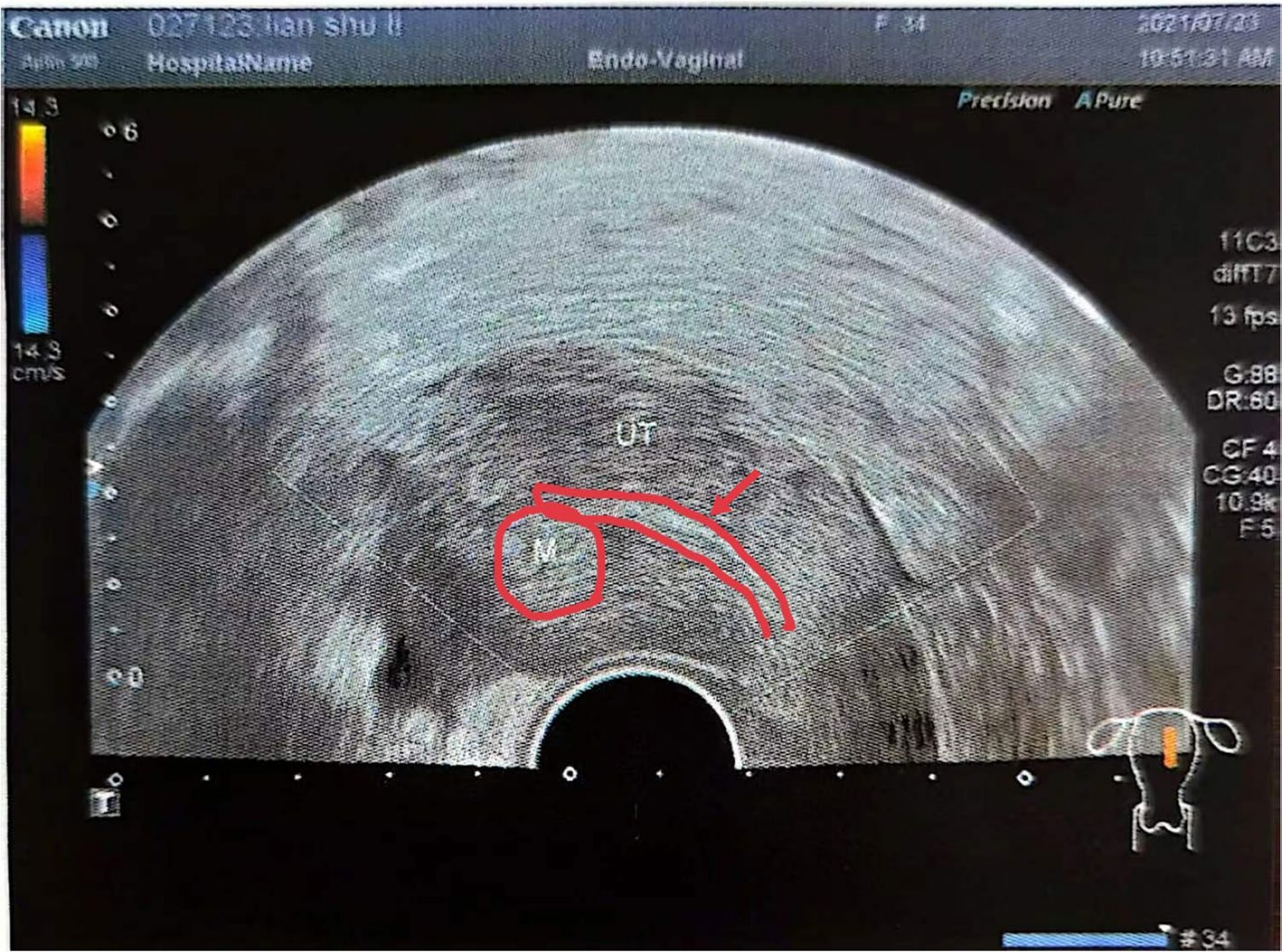
Sonographic presentation of type 3 myoma and the endometrium

**Fig. 2:**
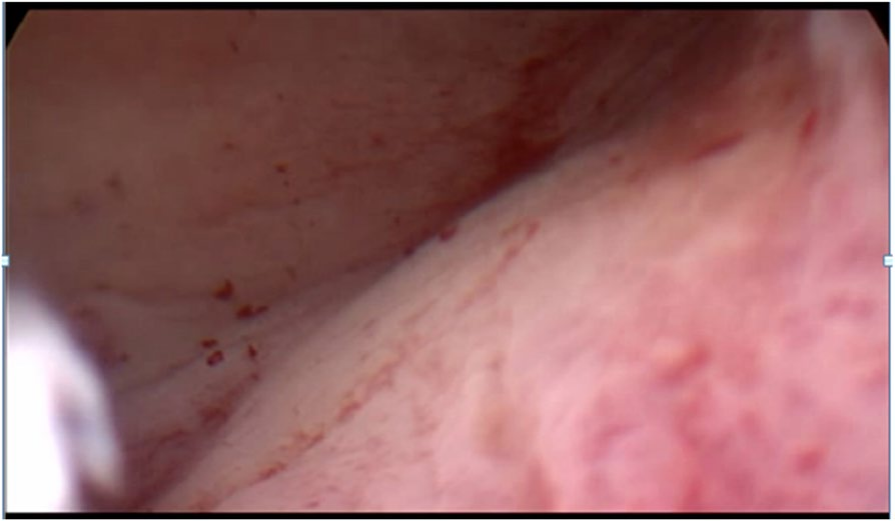
Hysteroscopic view that no alterations in the endometrial cavity

The International Federation of Gynaecology and Obstetrics (FIGO) developed staging scheme based on fibroid location including submucosal (SM), intramural (IM), subserosal (SS), and transmural lesions [8]. According to the FIGO fibroid classification system, IM fibroids include types 3, 4, and 5. Type 3 lesions are totally extra-cavitary but abut the endometrium [8]. By the use of transvaginal ultrasound (TVS), type 3 lesions were reported to distort the uterine endometrium, but hysteroscopy revealed no alterations in the endometrial cavity (Figs. 1, 2). Yan et al. explained that type 3 fibroids decreased the rates of implantation, clinical pregnancy and live birth in patients undergoing IVF, but did not significantly increase the clinical miscarriage rate in a retrospective study. Their findings showed that type 3 fibroids exert a negative impact on women with a single fibroid diameter (SD) or total fibroid diameter (TD) > 2.0 cm [9]. Rikhraj et al. reviewed 15 quantitative studies out of 139 identified records. They concluded that patients with non cavity-distorting intramural fibroids undergoing in vitro fertilization (IVF) have a 44% lower chance of a live birth and 32% lower odds of a clinical pregnancy than unaffected women [10].

The Practice Committee of the American Society for Reproductive Medicine (ASRM) issued guidelines stating that myomectomy may be considered to optimize the pregnancy outcomes for women with asymptomatic myomas. There is fair evidence that hysteroscopic myomectomy for asymptomatic cavity-distorting myomas improves the clinical pregnancy rate [11]. Howerer few studies have examined the effect of surgical removal of type 3 fibroids on the pregnancy outcomes. Hysteroscopic myomectomy is an effective treatment for type 3 fibroids. In a retrospective cohort study Capmas P et al. identified that hysteroscopic resection is a potential alternative to traditional surgery for type 3 myoma; however this procedure must be performed by skilled surgeons because of its difficult nature [12]. Howerer there has few study of the pregnancy outcomes in infertile women after hysteroscopic resection for type 3 fibroids to date.

Therefore, we performed a single-center, retrospective, case–control study to evaluate the effect of hysteroscopic resection of type 3 fibroids on the pregnancy outcomes in infertile women.

## Methods

### Study design

This retrospective case–control study was conducted at the Center for Reproductive Medicine, Tianjin Central Hospital of Gynaecology Obstetrics from January 1, 2014 to June 30, 2021, through the analysis of our electronic records. Before the treatment, all patients were required to undergo TVS and diagnostic hysteroscopy; more than one ultrasonography was performed to observe the change in the myoma before embryo transfer. All TVS and diagnostic hysteroscopic examinations were performed by professional clinicians. The inclusion criteria for the type 3 fibroid group and the hysteroscopic myomectomy group were as follows: 1) age ≤ 40 years at the time of commencement of IVF-ICSI treatment; 2) diagnosis of type 3 fibroids or accepted for hysteroscopic resection for type 3 fibroids; and 3) SD or TD > 2.0 cm. The following exclusion criteria were used: 1) oocyte donor treatment cycles; 2) presence of chromosomal abnormalities (including chromosome polymorphism); 3) history of other uterine surgery (including endometrial polyps, cesarean section, etc.); 4) presence of intracavitary lesions, including SM fibroids; 5) single fibroid > 5.0 cm; 6) cervical fibroids; 7) unclear ultrasound description of fibroids; 8) preimplantation genetic testing was performed; and 9) congenital or acquired uterine malformations.

The patients were divided into a type 3 fibroid group (nonsurgery group) and a hysteroscopic myomectomy group (surgery group) according to whether they underwent a hysteroscopic myomectomy. The control group in our study was selected from patients who were treated with IVF only because of fallopian tube factors. According to the age of the nonsurgery group and surgery group, random sampling was carried out in patients between 25 and 47 years old to determine a control group. The nonsurgery group and surgery group patients were assessed for the treatment of hysteroscopic myomectomy, according to each patient’s condition and their personal wishes. All patients underwent controlled ovarian stimulation (COS) using a routine protocol (long, short, GnRH antagonist protocol, or other protocol, including ultralong protocol, mini-stimulation protocol, natural protocol, etc.) at our centre. One experienced surgeon (who had performed more than 500 operative hysteroscopies per year for more than 10 years as a resident) performed all of the procedures.

### Outcomes

The outcomes measured in our retrospective study included the average transfer times to live birth(s), cumulative clinical pregnancy rate (CCPR), and cumulative live birth rate (CLBR). The clinical pregnancy rate was defined as the presence of a gestational sac seen on transvaginal ultrasound per embryo transfer cycle. The live birth rate was defined as the delivery of a live birth(s) (≥ 20 completed weeks of gestational age.) per embryo transfer cycle [13]. The CCPR was calculated as the first gestational sac observed and the CLBR was calculated as the live births achieved after all cycles of embryo transfer (cycles using fresh embryos and all frozen-thawed embryos) among both groups [14]. The analysis of the transfer and pregnancy outcomes of the patients in the hysteroscopic myomectomy group started from the first transfer after the operation. Furthermore, we conducted a self-controlled case series study of the surgery group to compare the transfer and pregnancy outcomes before and after the operations.

### Surgeries

Operative hysteroscopy was performed using a 26Fr hysteroscope equipped with a 30-degree lens (Endoskope; Karl Storz GmbH and Co., Tuttlingen, Germany). Saline solution (0.9%) (China Otsuka Pharmaceutical Co., Ltd.) was used as the distention medium with a fluid management system (Richard-Wolf, Germany). The dilatation pressure was 80-100 mmHg (1 mmHg = 0.133 kPa), and the electric power was 80w. All operations were performed under general anesthesia.

**Fig. 3:**
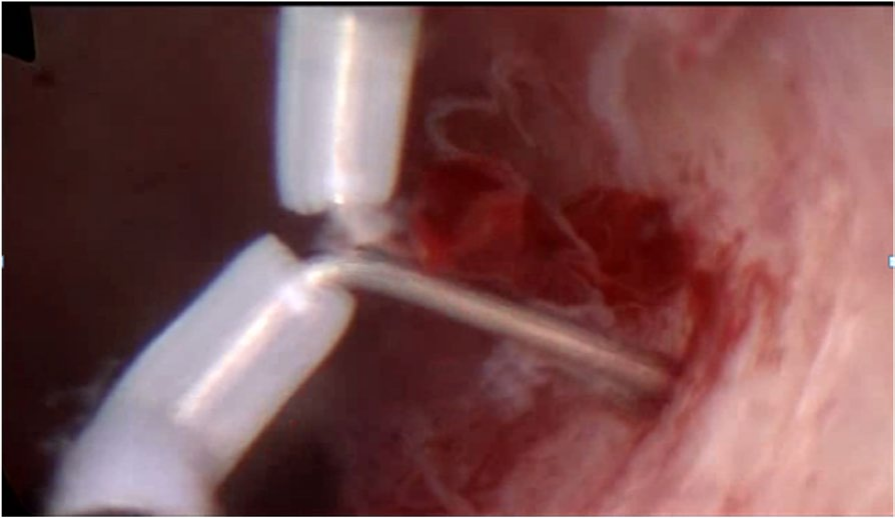
Reveal type 3 fibroid with needle electrodes longitudinally

**Fig. 4:**
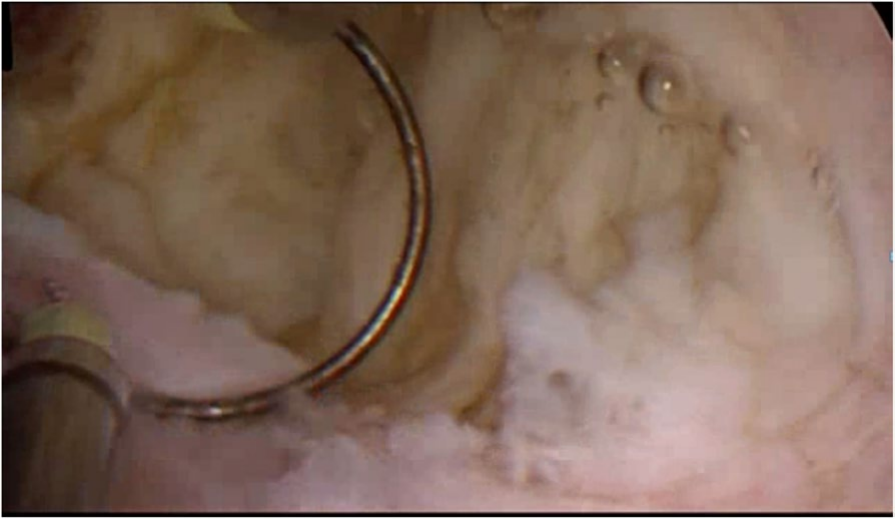
Loop resection of the myoma

**Fig. 5:**
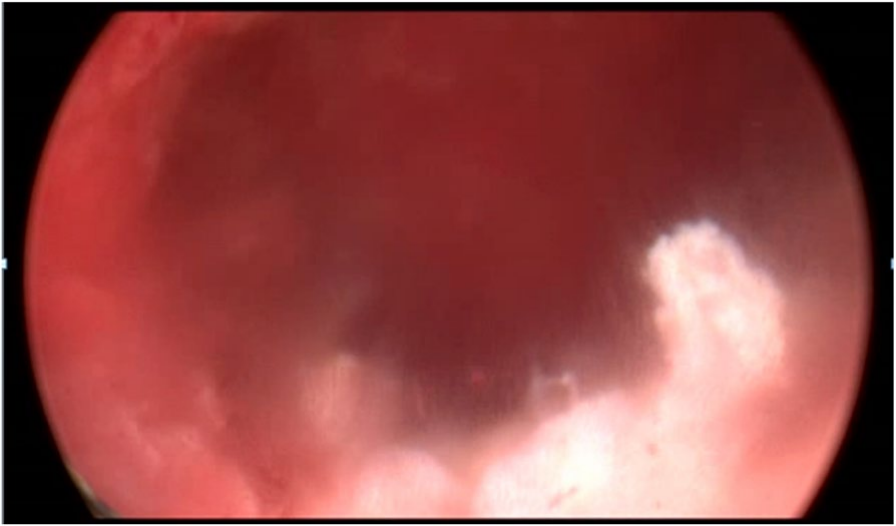
Hysteroscopic view at the end of resection

The surgeon searched for the location of the fibroids in combination with ultrasound examination, measured the thickness of the fibroids from the uterine serosa, and confirmed that they are type 3 fibroids again. Hysteroscopic resection for type 3 myoma began with an incision of the endometrium covering the myoma with bipolar energy (Olympus, Japan). Under ultrasound guidance, the uterine mucosa and myometrium were cut longitudinally with needle electrodes to reveal type 3 fibroids. Intravenous infusion of 10 IU oxytocin (Shanghai Harvest Pharmaceutical Co., Ltd.) in 500 ml of saline solution (0.9%) (China Otsuka Pharmaceutical Co., Ltd.) at a rate of 120 ml/h causes fibroids to bulge and the needle electrode was used to excise the tumor nucleus to avoid injury to the endometrium and normal muscle wall tissue. The excision was performed only on the tumor nucleus of the fibroid, and the pressure of the uterine distended uterus was gradually reduced to remove the type 3 fibroids completely (Figs. 3, 4, 5). At the same time, pay attention to the amount of fluid in and out to prevent complications such as hysteroscopic hyperhydration syndrome. The whole process of resection was carried out under ultrasound monitoring until ultrasound showed no fibroids remained, and the thickness of the tumor fossa from the uterine serosa was measured after surgery. If the diameter of the fibroids is large, the number is too big, or the location is not good enough to be removed at one time, two-stage surgery should be considered.

Postoperative, all patients received oral continuous combined 2 mg 17-β oestradiol and 10 mg dydrogesterone (Femoston®, Abbott Biologicals B.V.) for 2 cycles to promote the recovery of the endometrium. All of the women had a postoperative office hysteroscopy and TSV to control the completeness of the hysteroscopic resection after a rest period of 2 menstrual cycles after surgery.

### Statistical analysis

Statistical analysis was performed using SPSS 25.0. Student’s *t test*, the chi-square test, Fisher’s exact test, Kruskal–Wallis test, or the Mann–Whitney U test were used to compare the groups according to the data distribution. A *P value* of < 0.05 was considered to indicate statistical significance. Values are presented as the mean ± SD or median (lower quartile, upper quartile) according to the data distribution. Crude and adjusted odds ratios (ORs) with 95% confidence intervals (CIs) were calculated before and after adjusting for confounding variables, including age, infertility duration, body mass index (BMI), average endometrial thickness in the transfer cycle, and average high-quality embryo rate.

### Ethics statement

This study was approved by the Institutional Review Board (IRB) of Tianjin Central Hospital of Gynaecology Obstetrics (No: ZY2021010) and performed in accordance with the Helsinki Declaration. Written informed consent was obtained from the participants when they presented for IVF-ICSI treatment.

## Results

**Fig. 6:**
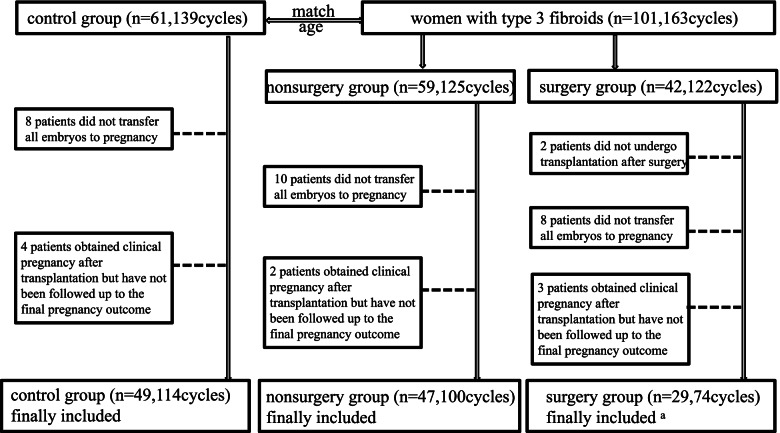
Flowchart of the study design. ^a^ Three patients who became pregnant naturally and delivered a live birth were included in the analysis of the pregnancy outcomes

According to the above inclusion and exclusion criteria, a total of 302 cycles of 162 women were enrolled in our study, including 125 cycles of 59 women with type 3 fibroids, 122 cycles of 42 women with hysteroscopic myomectomy, and 139 cycles of 61 control subjects. Among these 59 women with type 3 fibroids, seven suffered from multiple fibroids (five women had combined SS, two women had combined SS and multiple type 3 fibroids), and the average diameter of the type 3 fibroids was 2.45 cm. Among these 42 women with hysteroscopic myomectomy, six suffered from multiple type 3 fibroids but these had been removed by surgery (Fig. 6). There were no surgical complications in 42 patients. Hysteroscopy and ultrasound follow-up were performed 6–8 weeks after the operation. All patients had no residual fibroids, abnormal uterine bleeding or infection after the operation. Among them, only 2 patients had mild intrauterine adhesions and hysteroscopic adhesion separation surgery was performed.

The baseline characteristics of each group are presented in Table 1. There was no statistically significant difference in age, BMI, infertility duration, day 3 serum LH level, day 3 serum E2 level, or day 3 serum testosterone among the three groups. The day 3 serum FSH level was significantly lower in the control group than in the other two groups (*P* = 0.001). Table 2 shows the ovarian stimulation outcomes among the three groups. The number of MII oocytes retrieved was significantly higher in the control group. The number of cycles of fresh embryo transfer was significantly higher in the nonsurgery group than in the other two groups (*P* = 0.020), and the average transfer time to live birth(s) was significantly higher in the nonsurgery group than in the other two groups (*P* = 0.023).

**Table 1:** Baseline characteristics

Item	Control (*n* = 61)	Nonsurgery (*n* = 59)	Surgery (*n* = 42)	*P* value
Age, y	32.85 ± 4.20	34.73 ± 4.69	33.41 ± 4.24	0.062
< 37 years	43(75.4)	44 (76.6)	32 (76.2)	0.983
≥ 37 years	14 (24.6)	15 (25.4)	10 (23.8)	
Infertility duration, y	5.05 ± 3.23	5.19 ± 4.02	4.21 ± 2.55	0.325
BMI, kg/m2	23.05 ± 3.05	23.09 ± 3.17	22.43 ± 3.07	0.538
Basal FSH, mIU/L	6.13 ± 1.55	7.46 ± 2.29^a^	7.37 ± 2.33^b^	0.001
Basal LH, mIU/L	4.51 ± 6.21	4.00 ± 2.46	4.21 ± 1.74	0.499
Basal E_2_, ng/L	47.82 ± 21.17	48.94 ± 22.00	47.62 ± 18.32	0.941
Basal testosterone, ng/L	40.10 ± 16.22	36.23 ± 16.89	35.34 ± 17.39	0.309

Values are presented as the mean standard deviation or n (%)

*BMI* Body Mass Index, *FSH* Follicle Stimulating Hormone, *LH* Luteinizing Hormone, *E*_*2*_ oestradiol

^a^ shows that there were significant differences between the nonsurgery group and the control group

^b^ shows that there were significant differences between the surgery group and the control group

**Table 2:** Ovarian stimulation outcomes in three groups

Item	Control	Nonsurgery	Surgery	*P* value
Gn initial dosage, IU	273.31 ± 67.41	286.84 ± 95.44	293.31 ± 82.89	0.471
Duration of Gn stimulation, d	8.98 ± 2.01	9.39 ± 2.55	8.59 ± 2.25	0.254
Total dosage of Gn per cycle, IU	2438.92 ± 788.71	2807.23 ± 935.16	2654.73 ± 811.35	0.065
Average transfer endometrial thickness, mm	9.67 ± 1.52	9.64 ± 2.17	10.17 ± 1.96	0.944
Oocytes MII retrieved, n	19.92 ± 7.12^a^	9.47 ± 6.32^b^	12.32 ± 9.19^c^	0.000
High-quality embryo rate	39.10 ± 26.10	35.12 ± 29.74	36.40 ± 28.72	0.740
Cycle of transfer				0.020
Fresh embryo transfer cycle	30 (28.0)	43 (44.8)^a^	19 (27.9)^b^	
Frozen-thawed embryo transfer cycle	77 (72.0)	53 (55.2)	49 (72.1)	
Protocol of ovary stimulation				0.539
Short protocol	3 (4.9)	4 (6.8)	4 (9.5)	
Long protocol	40 (65.6)	33 (55.9)	27 (64.3)	
GnRH-A protocol	18 (29.5)	19 (32.2)	10 (23.8)	
Natural-cycle protocol	0 (0)	3 (5.1)	1 (2.4)	
Endometrium preparation protocol				0.719
Natural-cycle	64 (83.1)	42 (79.2)	38 (76.7)	
HRT	13 (16.9)	11 (20.8)	11 (22.4)	
Patients undergoing embryo transfer				0.332
Cleavage transfer rate	98(91.6)	88(91.7)	58 (85.3)	
Blastocyst transfer rate	9 (8.4)	7 (7.3)	10 (14.7)	
Sequential transfer rate	0 (0)	1 (1.1)	0 (0)	
High-quality embryo transferred rate	84 (78.5)	65 (67.7)	46 (67.6)	0.153
No. of embryos transferred, n	1.77 ± 0.47	1.74 ± 0.57	1.66 ± 0.51	0.415
Average transfer times to live birth, times	1 (1,2)	2 (1,2)	1 (1,1)^c^	0.023

Values are presented as the mean standard deviation, n (%) or median (lower quartile, upper quartile)

*Gn* Gonadotropin, *GnRH-A* Gonadotropin Releasing Hormone Antagonist, *HRT* Hormone Replacement Treatment cycle

^a^ shows that there were significant differences between the nonsurgery group and the control group

^b^ shows that there were significant differences between the surgery group and the control group

^c^ shows that there were significant differences between the surgery group and the nonsurgery group

To ensure the stability of the outcomes, the patients who had not achieved a pregnancy outcome by the statistical date were excluded from the analysis of the pregnancy outcomes in our study. The main pregnancy outcomes in the three groups are presented in Table 3. After adjusting for confounding factors among the three groups, the frequency of cumulative live births in the nonsurgery group was significantly lower than that in the control group (55.3% *vs.* 79.6%), with an OR of 0.316 (95% CI 0.114–0.880; *P* = 0.032). Compared with the control group, the surgery group patients had no statistically significant differences in the CCPR and CLBR.

**Table 3:** Binary logistic regression analysis of the pregnancy outcomes in the three groups

		Cumulative clinical pregnancy		Cumulative live birth	
Nonsurgery vs control	Rate	30/47 (63.8) vs 40/49 (81.6)		26/47 (55.3) vs 39/49 (79.6)	
	Crude OR	0.363 (0.143–0.921)	0.033	0.219 (0.118–0.717)	0.007
	Adjusted OR^a^	0.607 (0.198–1.863)	0.328	0.316 (0.114–0.880)	0.032
Surgery vs control	Rate	18/29 (62.1) vs 40/49 (81.6)		18/29 (62.1) vs 39/49 (79.6)	
	Crude OR	0.405 (0.141–1.167)	0.094	0.462 (0.163–1.305)	0.145
	Adjusted OR^a^	0.238 (0.059–1.103)	0.055	0.294 (0.083–1.044)	0.063
Surgery vs nonsurgery	Rate	18/29 (62.1) vs 30/47 (63.8)		18/29 (62.1) vs 26/47 (55.3)	
	Crude OR	0.927 (0.356–2.416)	0.877	1.322 (0.514–3.410)	0.563
	Adjusted OR^a^	0.388 (0.092–1.645)	0.199	0.667 (0.172–2.586)	0.558

^a^ Logistic regression analysis was conducted by adjusting for age, infertility duration, body mass index (BMI), average endometrial thickness in the transfer cycle and average high-quality embryo rate

### Sensitivity analysis

To restrict bias in the results, all the included patients who had not achieved a pregnancy outcome by the statistical date were analysed by the regression equation for sensitivity analysis, and the lowest and highest CLBRs were predicted. In this part, the patients who had not achieved a pregnancy outcome by the statistical date were supposed to have had live birth(s) or had live birth(s) to calculate the lowest and highest CLBR. The outcomes of the sensitivity analyses are presented in Table 4. After adjusting for confounding factors among the three groups, the frequency of the highest CLBR in the nonsurgery group was significantly lower than that in the control group (64.4% *vs.* 83.6%), with an OR of 0.305 (95% CI 0.136–0.900; *P* = 0.040). Compared with the control group, the surgery group patients showed no statistically significant differences in the highest CLBR.

**Table 4:** Sensitivity analysis

		Cumulative live birth lower limit		Cumulative live birth upper limit	
Nonsurgery vs control	Rate	26/59 (44.1) vs 39/61 (63.9)		38/59(64.4) vs 51/61(83.6)	
	Crude OR	0.444 (0.214–0.925)	0.030	0.355 (0.150–0.840)	0.019
	Adjusted OR^a^	0.451 (0.199–1.102)	0.050	0.350 (0.136–0.900)	0.04
Surgery vs control	Rate	18/42 (42.9) vs 39/61 (63.9)		31/42(73.8)vs 51/61(83.6)	
	Crude OR	0.423 (0.189–0.945)	0.036	0.553 (0.210–1.451)	0.229
	Adjusted OR^a^	0.292 (0.113–1.059)	0.043	0.563 (0.187–1.692)	0.332
Surgery vs nonsurgery	Rate	18/42 (42.9) vs 26/59 (44.1)		31/42(73.8)vs 38/59(64.4)	
	Crude OR	0.952 (0.428–2.115)	0.904	1.557 (0.652–3.718)	0.318
	Adjusted OR^a^	0.618 (0.230–1.663)	0.341	1.472 (0.481–4.500)	0.498

Data are presented as n (%) and odds ratios (95% confidence intervals)

*CI* Confidence Interval, *OR* Odds Ratio

^a^ Logistic regression analysis was conducted by adjusting for age, infertility duration, body mass index (BMI), average endometrial thickness in the transfer cycle and average high-quality embryo rate

In addition, there were seven patients who had undergone embryo transfer before and after surgery in the surgery group. The transfer and pregnancy outcomes of the seven patients are presented in Table 5. The transfer times to live birth, cumulative pregnancy rate, and cumulative live birth rate showed no statistically significant difference before and after the operations.

**Table 5:** Analysis of the transplantation and pregnancy outcomes in the hysteroscopic myomectomy group patients who underwent transfer before and after the operation

Item	Before	After	*P* value
Average transfers to live birth	2 (1,3)	1 (1,1)	0.165
CCPR	2/7 (28.6)	3/7 (42.9)	0.500
CLBR	1/7 (14.3)	3/7 (42.9)	0.280

Values are presented as n (%) or median (lower quartile, upper quartile)

*CCPR* Cumulative Clinical Pregnancy Rate, *CLBR* Cumulative Live Birth Rate

## Discussion

Type 3 fibroids are a special subtype of IM fibroid that are likely to affect the pregnancy outcomes of assisted reproductive techniques. They can affect the endometrial receptivity and the hormonal milieu, impede endometrial blood flow and alter the endometrial development which may be underlying causes of primary or secondary infertility [15, 16]. All published studies and meta-analyses reviewed by Donnez and Dolmans concur that non cavity-distorting intramural myomas do indeed have a deleterious impact on IVF outcomes [7]. Two factors have emerged as key factors: the fibroid size (the larger, the more TGF-b3 secretion) and proximity to the uterine cavity. In other words, a type 3 myoma of 2 cm or more will have a detrimental effect close to the endometrial lining. Hysteroscopic resection is a treatment for type 3 fibroids, but there has been no specific study of its efficacy to date. Moreover, we did not know whether hysteroscopic myomectomy could improve the fertility and IVF-ICSI outcomes of infertile women with type 3 fibroids. Our study focused on the effectiveness of hysteroscopic resection of type 3 fibroids on pregnancy outcomes in infertile women.

To exclude the influence of other infertility factors on the pregnancy outcomes, the study selected patients with tubal factors as the control group. The day 3 serum FSH level was significantly lower and the number of MII oocytes retrieved was significantly higher in the control group than in the other two groups. Therefore, the characteristics of the control group could have good ovarian function and better pregnancy outcomes. The current study demonstrates that type 3 fiboids significantly reduce the CLBR of IVF patients and increase the transfer times to live birth. The CLBR in the nonsurgery group was significantly lower than that in the control group. The CCPR and CLBR between the surgery and control groups were not statistically significant. The nonsurgery group had significantly higher average transfer times to live birth than the other two groups. Hysteroscopic myomectomy could improve the time to live birth to a certain extent, although, before and after hysteroscopic myomectomy, the average transfer times to live births, CCPR and CLBR were not significantly different. This may be related to the small number of patients, but compared with the two groups, the average transfer times to live birth after surgery were less than those of the preoperative group [1(1,1) *vs.* 2(1,3)]. The CCPR and the CLBR were higher than the preoperative group at 42.9% *vs.* 28.6% and 42.9% *vs.* 14.3%. In addition, three postoperative patients who obtained a spontaneous pregnancy and a live birth were followed up. Therefore, hysteroscopic resection could improve the clinical pregnancy rate and live birth rate of patients with type 3 fibroids, and shorten the time to live birth.

Several investigators have recommended that if the negative effect is related to the myoma size and proximity of the uterine cavity, why not try a medical approach to reduce the size of the myoma and to push it back deeper into the myometrium. Some studies found that the efficacy of drug therapy is uncertain, on the other hand, the treatment cycle is long (3–6 months) and the patient’s liver function needs to be monitored. The long-term use of these medications can cause osteoporosis, vasomotor and other symptoms of menopause [17, 18].

Open or laparoscopic myomectomy changed the fertility of infertile patients, however they had a high incidence of postoperative pelvic adhesions that can lead to pain and infertility, blood loss and a longer operating room time [6]. At the same time open surgery is invasive and the incision may be painful after surgery. In severe cases the wound may not heal properly. Laparoscopic removal of intramural fibroids results in uterine scarring which may affect subsequent pregnancies [6]. In contrast operative hysteroscopy is the optimal treatment for normalizing the uterine cavity in patients with lesions associated with infertility [6]. When a type 3 myoma is removed hysteroscopically using a bipolar loop electrode the surgery is contained within the pseudocapsule and no normal myometrium is damaged. Because there are no abdominal incisions with the hysteroscopic approach, the patient can return to normal activities in 24 h [6]. Unfortunately, it can also lead to complications such as the formation of intrauterine adhesions postoperatively. Postoperative intrauterine adhesions can occur in up to 40% of patients having myomectomies [19].

In our hospital and unit, TVS and hysteroscopy are performed by experienced clinicians, so the reliability of the data can be guaranteed. In this study the operation was monitored by ultrasound to reduce the risk of residual fibroids and uterine perforation. Ivan Mazzon et al.reported that cold loop hysteroscopic myomectomy is a safe and effective procedure that seems to be associated with a lower rate of intrauterine adhesions in comparison with the reported literature. However, this research was limited to G1 (submucous myoma with intramural extension less than 50%) and G2 (submucous myoma with intramural extension higher than 50%) myomas [20]. In this study, only the needle electrode was used for making the longitudinal incision of the endometrium to expose the fibroids and the larger fibroids were incised crosswise to minimize the endometrial damage. Intravenous infusion of oxytocin to bulge and the needle electrode was used to excise the tumor nucleus to avoid injury to the endometrium and normal muscle wall tissue. After the operation, the patients took two courses of Femoston orally to promote the recovery of the endometrium. In this study, the length of time between diagnosis and surgery was quite short. On the other hand we let each patient go through two menstrual cycles before returning for re-examinations after surgery, since the endometrial recovery time after hysteroscopy is 6–8 weeks [12]. All of the women had a postoperative inpatient hysteroscopy and ultrasound follow-up to control the completeness of the hysteroscopic resection. None of the patients had residual fibroids, abnormal uterine bleeding or infection. Only two patients had mild intrauterine adhesions and hysteroscopic adhesion separation surgery was performed. Hysteroscopic resection of type 3 fibroids can be an effective treatment for these patients.

In this study, we used a series of matching and exclusion criteria to minimize the interference factors. The COS protocols can affect the IVF success and there was no statistically significant difference in the ovarian stimulation protocols among the three groups [9]. As previously reported in the literature, type 3 fibroids with SD or TD > 2.0 cm can significantly decrease the live birth rate after IVF-ICSI, so we chose patients with SD or TD fibroids > 2.0 cm and single fibroids < 5.0 cm as the research subjects [9]. To select the appropriate research population and exclude the influence of other infertility factors, we selected patients who were treated with IVF only because of fallopian tube factors as the control group. The confounding factors in the observational studies were reasonably controlled. The follow-up time of the patients was 6 months to 2 years and 6 months (median follow-up time was 18 months), which ensured that the follow-up of the pregnancy outcomes of the patients was relatively complete.

The principal limitation of this study was the small number of patients, which led to a low statistical power. However, few women with type 3 myoma undergo hysteroscopic myomectomy. The sensitivity analysis suggests that the estimated value of the included study was basically within the confidence interval of the total effect size; that is, the result was stable. Selection bias in the data analysis is inevitable in a retrospective study. In addition, some patients underwent surgery after COS, and the stimulation of COS on fibroids cannot be ruled out. Therefore, randomized controlled long-term observational research with large sample sizes are suggested. It may be necessary to cooperate with multiple centres and combine basic research to expand the research on the diagnosis and treatment of type 3 fibroids, and ultimately better guide clinical work in the future.

To the best of our knowledge, this is the first independent study to address the impact of hysteroscopic resection of type 3 fibroids on the pregnancy outcomes of infertile women. Furthermore, this study shows the necessity of a clinical consultation with infertile patients with type 3 fibroids with SD or TD > 2.0 cm regarding the need for hysteroscopic myomectomy. One of the important advantages of the present study was the use of multiple matching criteria and randomization principles to minimize the clinical heterogeneity of the included patients.

## Conclusion

Type 3 fibroids significantly reduce the cumulative live birth rate of IVF patients. Ultrasound-guided hysteroscopic myomectomy can be used as a treatment for type 3 fibroids, and could improve the cumulative pregnancy rate, cumulative live birth rate, and the time to reach live birth of infertile patients with type 3 fibroids.

## Data Availability

The data used or analyzed during the current study are included within the article. The datasets are not publicly available due to the hospital policy and personal privacy. However, the datasets are available from the corresponding author on reasonable request.

## Abbreviations

FIGO: The International Federation of Gynecology and Obstetrics
IVF: In vitro Fertilization
ICSI: Intracytoplasmic sperm injection
SM: Submucosal
IM: Intramural
SS: Subserosal
TVS: Transvaginal ultrasound
SD: Fibroid diameter
TD: Total diameter
ASRM: Practice Committee of the American Society for Reproductive Medicine
COS: Controlled ovarian stimulation
ORs: Odds ratios
CIs: Confidence intervals
BMI: Body mass index
IRB: Institutional Review Board
FSH: Follicle stimulating hormone
LH: Luteinizing hormone
E2: Estradiol
HRT: Hormone replacement treatment cycle
Gn: Gonadotropin
GnRH: Gonadotropin releasing hormone
CCPR: Cumulative clinical pregnancy rate
CLBR: Cumulative live birth rate
